# Recognition of Significantly Delayed Spinal Cord Ischemia Following Thoracic Endovascular Aortic Repair: A Case Report and Review of the Literature

**DOI:** 10.7759/cureus.51522

**Published:** 2024-01-02

**Authors:** Hannah Kelly, Danielle Herman, Kiana Loo, Adeeb Narangoli, Emily Watson, Corey Berlant, Mina Huerta, Collin M Labak, Xiaofei Zhou

**Affiliations:** 1 Neurology, Case Western Reserve University School of Medicine, Cleveland, USA; 2 Neurology, University Hospitals Cleveland Medical Center, Cleveland, USA; 3 Emergency Medicine, University Hospitals St. John Medical Center, Westlake, USA; 4 Neurological Surgery, University Hospitals Cleveland Medical Center, Cleveland, USA

**Keywords:** thoracic endovascular aortic repair, tevar, spinal cord ischemia, map augmentation, lumbar spinal drain, csf diversion

## Abstract

Spinal cord ischemia (SCI) is an uncommon but serious complication of thoracic endovascular aortic repair (TEVAR). SCI after TEVAR is thought to result from decreased segmental blood supply to an important network of collateral blood flow in the spinal cord. Little is known about the prevalence and optimal treatment of SCI that occurs beyond the periprocedural period. We report a case of delayed SCI in a 67-year-old patient who underwent TEVAR. The patient presented almost two years after TEVAR with acute paraplegia preceded by pre-syncope. The delayed SCI was likely triggered by pre-syncope, a thrombosed endoleak shown on imaging, and the patient's vascular risk factors. Treatments included cerebrospinal fluid (CSF) drainage, mean arterial pressure (MAP) augmentation, and a naloxone infusion, which resulted in moderate recovery in lower extremity motor function. This case highlights the tenuous nature of spinal cord perfusion after TEVAR and that prompt recognition and early treatment of SCI are critical in preventing the progression from ischemia to infarction.

## Introduction

Spinal cord ischemia (SCI) is a disabling condition that results from hypoperfusion of the spinal cord. Depending on the level of spinal cord involvement, it presents as new-onset upper and/or lower extremity sensorimotor deficits, urinary incontinence, and/or bowel incontinence [1]. Common causes of SCI include atherosclerotic disease, post-traumatic injury, or idiopathic disruption of blood supply to the spinal cord. SCI often occurs with surgical or endovascular repairs of aortic dissections or aneurysms and holds a one-year mortality of up to 75% [2]. Recent studies show a perioperative incidence of SCI between 0% and 35% for thoracic endovascular aortic repair (TEVAR) and open surgical repair [3]. Pre-existing conditions, such as hypertension, extensive atherosclerosis, renal failure, and chronic obstructive pulmonary disease (COPD), place patients at increased risk for SCI post-TEVAR. Perioperative or postoperative risk factors include the following: concomitant or prior aortic interventions, hypotension, use of three or more stents, long segment aortic involvement, use of an iliac conduit, and coverage of either the artery of Adamkiewicz or two or more vascular territories, such as the hypogastric, left subclavian, intercostal, lumbar, or internal iliac arteries during TEVAR [4-6].

Existing strategies for preventing SCI post-aortic repair encompass elevating blood pressure, cerebrospinal fluid (CSF) diversion through lumbar spinal drain placement, and optimizing hemoglobin and oxygen delivery [7]. There are other interventions under investigation, such as an intraoperative bypass for distal perfusion, deep hypothermic circulatory arrest, selective intercostal artery reimplantation, staged repairs, spinal cord preconditioning with minimally invasive segmental artery coil embolization, and perioperative medications such as naloxone, mannitol, corticosteroids, and intrathecal papaverine. SCI after TEVAR generally occurs in the immediate periprocedural setting; however, some cases reported in the literature describe delayed SCI after such repairs [3]. Thus, early identification of SCI in this setting is important to minimize the risk of permanent neurologic deficits. Here, we present a case of SCI in a patient who underwent TEVAR two years prior to highlight the importance of recognizing and promptly treating delayed SCI.

## Case presentation

**Figure 1:**
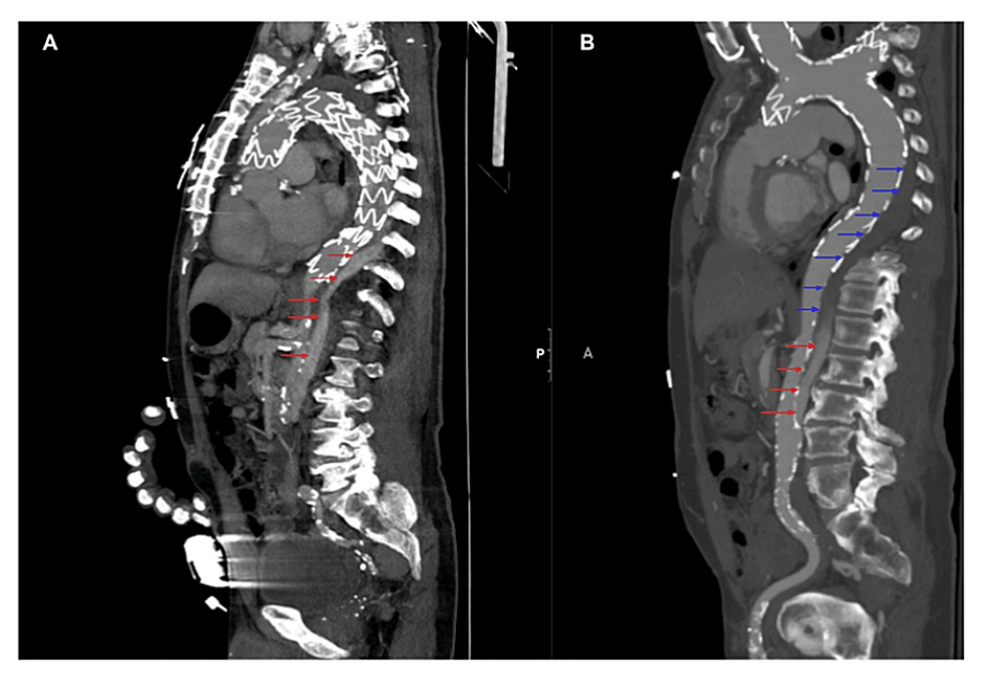
Aortic endoleak and subsequent endoleak progression and thrombosis (A) Computed tomography angiogram (CTA) of the chest/abdomen/pelvis demonstrating an endoleak with filling throughout the false lumen (red arrows) from a prior admission. (B) CTA chest/abdomen/pelvis from the patient’s presented admission showing further propagation of the endoleak compared to in A (red arrows). There is an absence of contrast within the dilated false lumen (blue arrows), suggesting interval thrombosis of a large portion of the endoleak from about T5-T8.

The patient is a 67-year-old male with a past medical history significant for hypertension, hyperlipidemia, COPD, stage II chronic kidney disease, obstructive sleep apnea, benign prostatic hyperplasia, prior right midbrain ischemic infarct (with no residual deficits), and Stanford type A aortic dissection. Relevant home medications at the time of presentation included amlodipine 5 mg, atorvastatin 80 mg, metoprolol succinate 100 mg, and prazosin 1 mg. He underwent an emergent open aortic dissection repair in 2019 with persistent aneurysmal expansion. Ultimately, he required an aortic debranching and TEVAR in 2021. No surgical complications were reported. Postoperatively, the patient was managed with a lumbar spinal drain and mean arterial pressure (MAP) augmentation with no neurologic deficits reported. TEVAR was complicated by a type 1A endoleak noted approximately four months postoperatively (Figure 1A).

Nineteen months after TEVAR, the patient presented to the emergency department with rapidly progressive paraplegia and numbness of his abdomen and lower extremities. He reported being in his usual state of health when he experienced non-traumatic, sudden-onset presyncope. This was followed by a sensation of his legs “going limp” along with numbness of his abdomen and lower extremities, causing him to fall from a seated position.

Physical exam was notable for diminished strength in his bilateral lower extremities (Table 1), as well as reduced sensation to light touch, vibration, and pain below the T8 distribution. Computed tomography (CT) of the brain without contrast showed no acute intracranial pathology. Computed tomography angiography (CTA) of the chest, abdomen, and pelvis demonstrated progression of the aortic endoleak with a new paucity of contrast within the dissection flap suggestive of interval thrombosis of a large portion of the endoleak from about T5-T8 (Figure 1B).

**Table 1:** Timeline of interventions IV: intravenous, gtt: drip, RLE: right lower extremities, LLE: left lower extremities, MAP: mean arterial pressure

Interventions					IV naloxone gtt						IV naloxone gtt		IV naloxone gtt	
			Lumbar drain		Lumbar drain		Lumbar drain		Lumbar drain		Lumbar drain		Lumbar drain	
			MAP>100		MAP>100		MAP>100		MAP>100		MAP>100		MAP>100	
	Day 0		Day 1		Day 2		Day 3		Day 4		Day 5		Day 6	
	LLE	RLE	LLE	RLE	LLE	RLE	LLE	RLE	LLE	RLE	LLE	RLE	LLE	RLE
Hip flexion	1	0	1	1	2	2	2	2	4	3	2	2	3	4-
Knee extension	1	0	1	1	4	3	4	3	4	3	3	3	3	4-
Plantar flexion	1	2	3	2	4	2	4	4	5	4	5	4	4+	5
Dorsiflexion	1	2	3	2	3	2	4	4	5	4	5	4	4+	5

**Figure 2:**
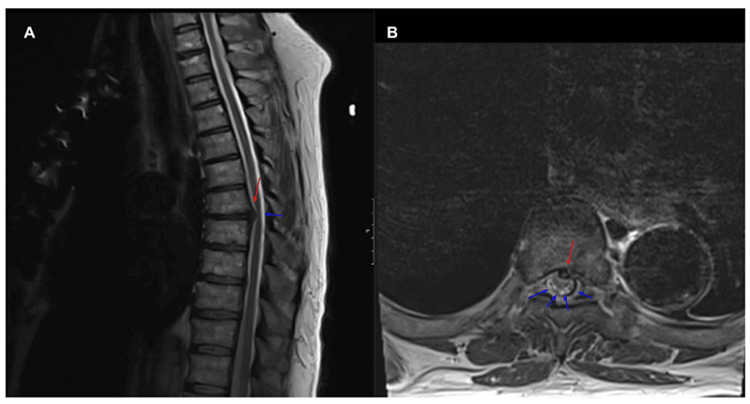
Thoracic spinal cord MRI Sagittal (A) and axial (B) T2-sequences of a thoracic spinal cord magnetic resonance imaging (MRI) without contrast demonstrating a disc herniation at the T7-8 interspace abutting the spinal cord eccentrically on the left (red arrow). There is space within the thecal sac seen dorsally (blue arrows).

**Figure 3:**
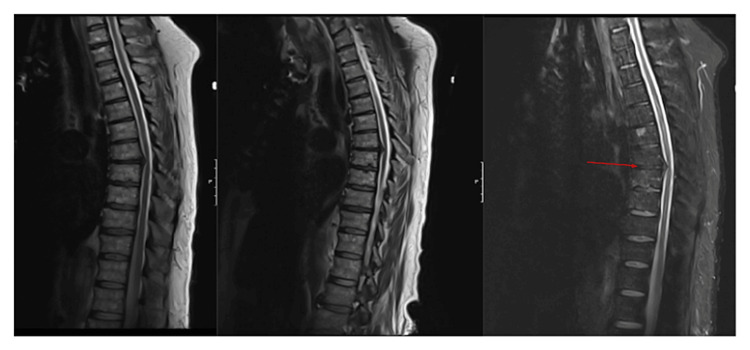
MRI of the thoracic spine in flexion T2-sequences of a MRI thoracic spine without contrast in a neutral (left) and with the patient in flexion (center) showing no dynamic compression on the spinal cord from the T7-8 disc herniation. Short T1 inversion recovery (STIR) sequences from this MRI (right) additionally show no edema within the intervertebral disc to suggest an acute herniation (red arrow).

Magnetic resonance imaging (MRI) of the thoracic spine showed the T7-T8 disc pressing against the spinal cord ventrolaterally without T2 signal changes or evidence of ligamentous injury on short T1 inversion recovery (STIR) images (Figure 2). The absence of STIR signal change suggested this disc herniation was likely chronic. MRI of the thoracic spine in flexion further indicated that the T7-T8 disc was not causing significant cord compression (Figure 3).

**Figure 4:**
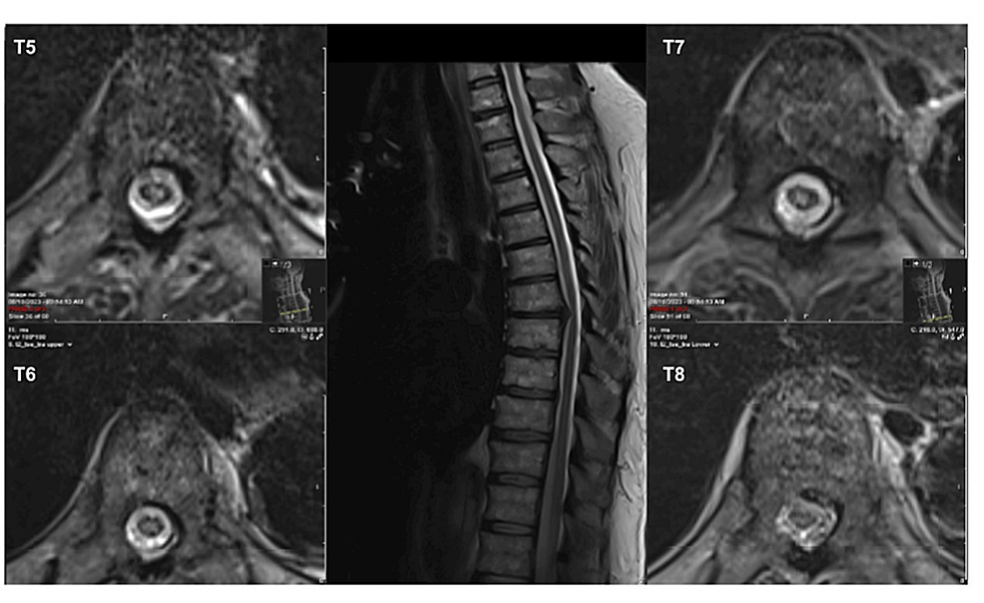
T2 sequences of an MRI thoracic spine Sagittal (center) and axial cuts through the mid-vertebral body at various levels (labeled) on post-injury day four demonstrating T2 signal change throughout the spinal cord, suggestive of an ischemic event.

Given the sudden onset of symptoms and history of TEVAR, this clinical presentation was concerning for SCI. The decision was made to augment MAP and place a lumbar spinal drain to optimize spinal cord perfusion (Figure 4). The lumbar drains at our institution do not have the capacity for pressure transduction. With the patient’s baseline hypertension and presentation MAP of 90 mmHg, a MAP goal of greater than 100 mmHg was set. Following placement of the lumbar spinal drain, the patient partially recovered sensory and motor function (Table 1). A naloxone infusion was also started on post-injury day two to enhance cord perfusion at the recommendation of the vascular surgery team (Table 1, Figure 4).

A second delayed MRI was completed on post-injury day four, including a T2 sequence in flexion to confirm there was no dynamic compression from disc herniation (Figure 3). T2 imaging also showed hyperintensities within the spinal cord spanning from T5 to T8, consistent with an ischemic event (Figure 4). Given significant artifacts, diffusion restriction in the spinal cord could not be assessed using the diffusion-weighted images from the initial and delayed MRIs. All therapies were continued through post-injury day six, at which time the patient’s exam stabilized over a 48-hour period. Therapies were then weaned, and the patient was ultimately discharged to a spinal cord injury rehabilitation center in stable condition.

At the patient's one-month follow-up with the spinal cord injury physician, he was noted to be ambulating without any assistive device, but did have continued issues with neurogenic bladder.

## Discussion

Compared to open aortic aneurysm repairs, TEVARs avoid significant hemodynamic changes believed to contribute to perioperative SCI, including aortic cross-clamping and reperfusion injury [4]. However, TEVAR still carries a risk of SCI, which can cause permanent neurologic deficits, decrease long-term survival, and worsen quality of life [8]. SCI after TEVAR largely results from decreased segmental blood supply to an important network of collateral blood flow in the spinal cord, particularly from branches of the subclavian, intercostal (especially the artery of Adamkiewicz), and hypogastric arteries.

The degree of disruption to the collateral network is determined by the number of segmental arteries covered during TEVAR. The spinal cord then becomes reliant on the remaining segments for perfusion [9]. While this collateral network can maintain spinal cord perfusion [10], disturbances to this tenuous blood supply can contribute to delayed SCI [5]. For example, atheroembolism from aortic plaques dislodged during or after TEVAR can occlude these segmental vessels and contribute to SCI [10]. Postoperative endoleaks may create a collateral flow that maintains spinal cord perfusion. However, if these endoleaks are sealed, subsequent loss of collateral circulation may increase the risk of delayed SCI [11-12]. Here, the significant thrombotic progression of the patient’s endoleak (Figure 1) may be a potential etiology for the delayed presentation, given that the endoleak thrombosed at the same spinal cord levels involved in the ischemic event (T5-T8). While the aortic endoleak appeared to propagate and expand in size over time, a significant portion of the endoleak also likely thrombosed (Figure 1). The lack of collateral flow from the large thrombosed portion of the endoleak likely overcame any potential increase in collateral flow from the propagated portion, perhaps contributing to SCI.

**Figure 5:**
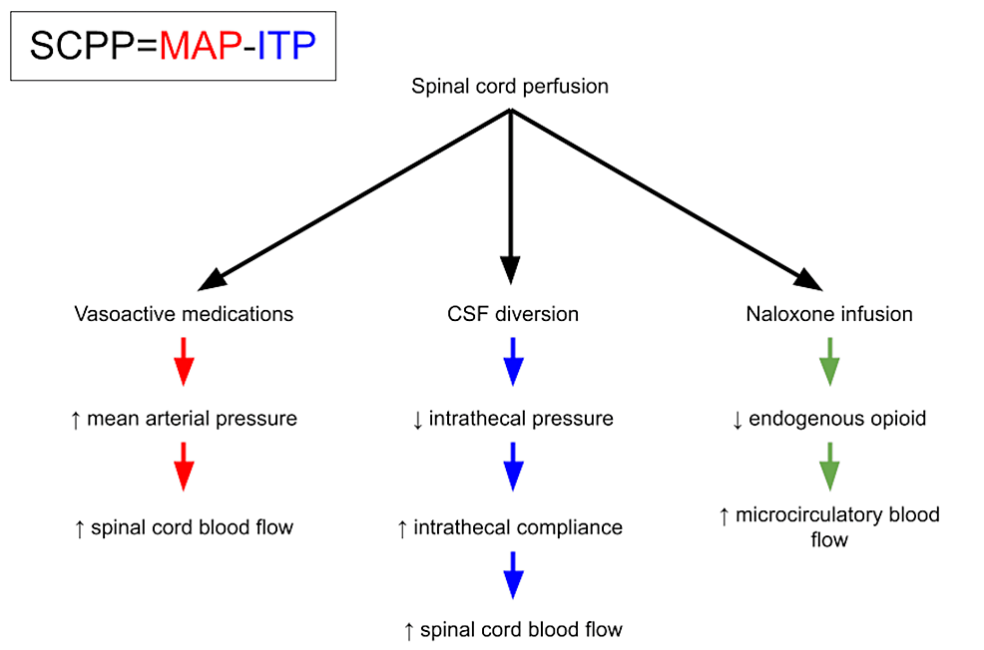
Flowsheet A flowsheet demonstrating the relationship between spinal cord perfusion pressure (SCPP), mean arterial pressure (MAP), and intrathecal pressure (ITP). The interventions employed for this patient are shown, including the proposed mechanisms of each therapy.

Regarding the management of post-TEVAR SCI, the U.S. Aortic Research Consortium recommends placing a CSF diversion device, such as a lumbar spinal drain, increasing CSF drainage up to 30 mL/hour, permissive hypertension to maintain MAP above 90 mmHg, and maintaining hemoglobin levels ≥10 mg/dL. Other measures, including perioperative naloxone infusions, were not routinely used by the group [13]. These interventions are based on the principle that spinal cord perfusion pressure is the difference between MAP and intrathecal pressure (Figure 5). Therefore, augmenting MAP with vasopressor medications and decreasing intrathecal pressure with CSF diversion can effectively increase spinal cord perfusion. While MAP augmentation and CSF diversion are generally well-tolerated, the risk of cardiovascular strain from prolonged vasopressor medications and the risk of spinal headache, bleeding, and brainstem herniation from lumbar spinal drains should be carefully considered [6].

Naloxone infusions have also been associated with a decreased incidence of SCI when combined with CSF diversion and/or other interventions [14,15]. In clinical studies, naloxone has been shown to reduce CSF concentrations of excitatory amino acids, such as glutamine, which is correlated with a lower incidence of SCI [16]. It is important to note that the link between naloxone and decreased risk of SCI remains unclear. This uncertainty should be weighed against the risk of suboptimal postoperative pain control [17].

The risk of SCI after TEVAR is the highest during the 30-day perioperative period; however, limited data exist on delayed SCI [5]. Prior reports have described cases of SCI six [18], eight [2,9], and 10 [5] months post-TEVAR in the setting of hypotension and anemia from gastrointestinal bleeding [2] and acute graft thrombosis [9]. These case reports highlight that hemodynamic instability at any point post-TEVAR may make patients vulnerable to SCI [5]. Patients with the previously mentioned risk factors have an increased risk of suffering SCI. In particular, patients with severe atherosclerotic disease and prior aortic surgery are at higher risk of delayed SCI because of baseline diminished collateral blood supply to the spinal cord [2]. Minimizing episodes of hypotension or decreased cardiac output is critical in post-TEVAR patients, as their spinal cord perfusion relies heavily on a precarious collateral network.

Our patient had several risk factors for post-TEVAR SCI, including hypertension, renal insufficiency, COPD, an open aortic dissection repair two years prior to TEVAR, an open aortic arch debranching at the time of TEVAR, and coverage of the left subclavian artery during TEVAR. Moreover, the onset of lower extremity weakness in our patient was preceded by presyncope. This history suggests hypotension may have precipitated the patient’s SCI despite being normotensive upon presentation. Interestingly, our patient also had evidence of an endoleak on imaging four months post-TEVAR and 10 months prior to this admission. Upon presenting with lower extremity weakness, CTA chest/abdomen/pelvis showed thrombosis of the endoleak. Losing this important collateral flow may have lowered the threshold for ischemia in our patient.

An interesting part of the patient's post-presentation management was the choice to increase MAP goals to over 100 mmHg rather than the recommended 90 mmHg. The patient was hypertensive at baseline, and baseline MAPs with no augmentation by vasoactive medications were 90 mmHg. We, therefore, decided that optimizing perfusion should be a moving target based on the patient’s baseline cardiac function and blood pressure. While no data exist on MAP goals above 100 mmHg in significantly delayed SCI after TEVAR, the target MAP is sometimes increased to 100-115 mmHg in the event of immediate or delayed paraplegia/paraparesis upon emergence from anesthesia [19]. This practice emphasizes that higher MAP goals are sometimes indicated to improve the likelihood of neurologic recovery. On the other hand, a prospective observational study of spinal cord injury patients found that maintaining spinal cord perfusion pressure above 50 mmHg is associated with greater neurologic recovery, while specific cutoffs for MAP or intrathecal pressure were not predictive. This suggests that examining spinal cord perfusion pressure can help guide blood pressure management and CSF diversion to optimize neurologic improvement in SCI [20].

Moreover, strict blood pressure control is not routinely recommended until four to six weeks after TEVAR to lower the risk of delayed SCI. Limited data exist on long-term hypertension management in these patients; however, it seems reasonable that less aggressive management may persist in the chronic setting, especially for patients with multiple risk factors for delayed SCI, as in the present case. This reinforces the importance of carefully monitoring blood pressure and maintaining parameters high enough to avoid SCI yet low enough to prevent cardiovascular strain in post-TEVAR patients [19].

Determining the etiology of our patient’s spinal cord injury was further complicated by the thoracic spine MRI showing an intervertebral disc abutting the spinal cord at the same level at which our exam localized the lesion. SCI was deemed most likely given the patient’s extensive vascular history, the prodrome of pre-syncope followed by acute paraplegia without trauma, and the lack of imaging findings consistent with acute disc herniation, ligamentous injury, or cord compression. The decision was made to proceed with medical management per our institution’s protocol.

It is important to mention that a spinal cord contusion at T8 from chronic degenerative disc disease is not out of the realm of possibility as a contributing factor to the patient’s lower extremity weakness. Nonetheless, the treatment for incomplete spinal cord injury due to contusion and cord ischemia remains the same. While spinal cord compression and ischemia can present similarly with acute sensorimotor deficits, compressive versus ischemic spinal cord injuries require different treatments, namely, surgery or medical management. In our case, spinal cord compression was appropriately considered in the differential diagnosis given the evidence of disc herniation, but it was ultimately deemed less likely given the imaging findings showing space within the thecal sac dorsally and a lack of dynamic compression of the spinal cord. This case, therefore, emphasizes the importance of considering clinical history and imaging findings when determining the mechanism of a spinal cord insult.

Limitations to this case report include biases inherent to a retrospective study design, limited possibility of generalizing the validity of the study, and an inability to parse out the differential impact of the three interventions used in our patient. The fact that our patient had multiple concomitant risk factors for delayed SCI (atherosclerotic risk factors, a thrombosed endoleak, and a hypoperfusion event), as well as evidence of degenerative disc disease at the same level of the spinal cord injury further complicates the ability to determine the specific etiology of SCI in this case.

## Conclusions

This case adds to the limited literature on delayed spinal cord ischemia after TEVAR. To our knowledge, we report the first use of a rescue naloxone infusion for delayed spinal cord ischemia after TEVAR. While the impact of individual rescue therapies cannot be parsed out, this case emphasizes the potential role of therapies in addition to CSF diversion and MAP augmentation in improving neurologic recovery even almost two years after TEVAR. Moreover, we report the longest time elapsed between TEVAR and the onset of neurologic symptoms. This argues that TEVAR patients remain at risk for spinal cord ischemia even well beyond the initial perioperative period. Clinicians should, therefore, maintain a high index of suspicion for spinal cord ischemia when faced with patients experiencing acute sensorimotor deficits at any point post-TEVAR. Prompt recognition and early treatment of spinal cord ischemia are critical in preventing the progression from ischemia to infarction. Furthermore, the fact that these patients remain pressure-dependent for spinal cord perfusion raises important considerations when approaching long-term blood pressure management in the outpatient setting.
